# The Incidence of Pregnancy-Associated Cushing's Disease and Its Relation to Pregnancy: A Retrospective Study

**DOI:** 10.3389/fendo.2020.00305

**Published:** 2020-05-29

**Authors:** Keyun Tang, Lin Lu, Ming Feng, Hanlin Zhang, Kang Chen, Xu Sun, Huijuan Zhu, Renzhi Wang, Zhaolin Lu

**Affiliations:** ^1^Key Laboratory of Endocrinology of National Health Commission, Department of Endocrinology, Peking Union Medical College Hospital, Chinese Academy of Medical Science and Peking Union Medical College, Beijing, China; ^2^Eight-Year Program of Clinical Medicine, Peking Union Medical College, Chinese Academy of Medical Science and Peking Union Medical College, Beijing, China; ^3^Department of Neurosurgery, Peking Union Medical College Hospital, Chinese Academy of Medical Science and Peking Union Medical College, Beijing, China

**Keywords:** Cushing's disease (CD), pituitary corticotroph adenoma, pregnancy outcome, peripartum, low birth weight

## Abstract

**Purpose:** Cushing's disease (CD) is one of the most severe endocrine disorders and primarily affects women of reproductive age. The peripartum period has been observed to be a common time to develop CD. This study aims to retrospectively analyze the clinical characteristics of CD potentially associated with pregnancy and to evaluate relevant pregnancy outcomes.

**Methods:** Patients who underwent surgery from January 2010 to May 2019 at Peking Union Medical College Hospital (PUMCH) with biochemically and pathologically confirmed CD were retrospectively analyzed. Pregnancy-associated CD was defined as CD onset during gestation or within 12 months after delivery or abortion. Data including demographics, biochemical tests, magnetic resonance imaging (MRI) findings, and disease outcomes were obtained from all patients by reviewing their medical records. Information regarding pregnancy was collected through a supplementary online questionnaire.

**Results:** In a series of female patients (*n* = 70) of reproductive age with childbearing desire, 27.1% (*n* = 19) met the criteria for pregnancy-associated CD. The timing of diagnosis of pregnancy-associated CD was 2.7 ± 3.4 years after symptom onset, and the overall remission rate for these women was 89.5%. Three patients with pregnancy-associated CD developed hypertension during pregnancy, two of whom had new-onset hypertension at 16 weeks of gestation and one of whom had a complication of severe diabetes. The rates of spontaneous abortion and preterm birth among the women with pregnancy-associated CD were 26.3 and 28.6%, respectively. The proportions of all low-birth-weight (LBW) newborns (*p* = 0.002) and term LBW newborns (*p* = 0.033) were significantly higher in the pregnancy-associated CD group than in the non-pregnancy-associated CD group.

**Conclusions:** In this study, a total of 27.1% of women of reproductive age with CD had pregnancy-associated CD, which might be induced by the hormonal milieu of pregnancy. An increased risk of having a LBW newborn was observed among mothers with pregnancy-associated CD. A high degree of clinical suspicion for CD may be warranted in the peripartum period. Patients with symptoms suspicious for CD throughout pregnancy and after childbirth, such as early-onset hypertension, severe hyperglycemia, and persistent weight gain, should be carefully diagnosed and closely monitored by clinicians.

## Introduction

Cushing's disease (CD) is a severe endocrinopathy caused by a pituitary corticotroph adenoma that primarily affects females, with a previous series noting a 1:1.5–15 male-to-female ratio and a high incidence of occurrence among females of childbearing age (1–3). CD occurs less frequently during pregnancy than during the non-pregnancy period, likely because hypercortisolism and hyperandrogenism caused by CD impaired hypothalamic-pituitary-gonadal function and regular ovulation (4). In a series of 195 females with pituitary-dependent Cushing's syndrome, 63% had menstrual irregularity (4). Hypercortisolism during pregnancy may result in a higher risk of maternal comorbidities, including hypertension, impaired glucose tolerance, and preeclampsia. The rates of fetal prematurity, fetal death (intrauterine death or spontaneous abortion), and preterm birth are also increased. Other fetal complications, such as infection, hypoglycemia, and respiratory distress, are also more likely to occur in their newborns (5–8). Thus, the early diagnosis and treatment of CD in pregnant women should be emphasized.

Normal pregnancy is considered as a state of physiological hypercortisolism due to an increased hypothalamic-pituitary-adrenal (HPA) axis function, with serum total and urine-free cortisol reported as two- to four-fold elevated across gestation (7, 9). This elevation may have several explanations, such as desensitization of ACTH secretion to cortisol negative feedback, a continuous secretion of CRH and ACTH from placenta superimposing upon pituitary ACTH production, and an increase in response of cortisol release (6). Pregnancy can induce or exacerbate hypercortisolism in pathological conditions (10), which is associated with aberrant expression of the LH/hCG receptor on a primary adrenocortical tumor or placenta-derived ACTH stimulation of the MC2 receptor on an adrenocortical adenoma. Other placenta-derived factors such as CRH, CRH-like peptides, and estrogens may also contribute to the exacerbation of hypercortisolism during pregnancy (11). In contrast to the confirmed relationship between reproductive hormone changes and hypercortisolism mentioned above, the association between pregnancy and CD has not been extensively studied. In a retrospective study, Palejwala et al. (12) observed that 26.8% of women (*n* = 11) of reproductive age with CD developed CD during gestation or <12 months following pregnancy; this was defined as pregnancy-associated CD. They proposed a hypothesis that an overloading dose of cortisol from an overactivated hypothalamic-pituitary-adrenal axis during pregnancy could promote tumorigenesis or stimulate a subclinical ACTH-dependent adenoma. Data addressing a timeline for CD onset in the context of a woman's childbearing history and regarding relevant pregnancy outcomes among different subgroups of women are quite limited.

This retrospective study evaluated the clinical characteristics of pregnancy-associated CD (CD with an onset during gestation or within 12 months after delivery or abortion) and its relationship to pregnancy outcomes by investigating additional details regarding maternal and fetal characteristics during a follow-up period, including both the prenatal and postpartum periods. Suggestions regarding the early diagnosis of CD and management of CD in pregnant women were also analyzed and discussed.

## Methods

### Subjects

Patients were included if they met the following criteria: (1) female sex, (2) reproductive age (between 13 and 50 years of age), (3) transsphenoidal pituitary surgery (TSS) performed at Peking Union Medical College Hospital (PUMCH) from January 2010 to May 2019, (4) confirmed diagnosis of CD, and (5) complete data collection about her medical and pregnancy history based on medical charts, questionnaire feedback, and direct contact. In our study, reproductive age was considered to be the time between menarche and menopause, defined as ages 13–50 years. The exclusion criteria were as follows: (1) male sex, (2) adolescent female aged below 13 years without previous menarche, (3) menopausal female, (4) questionable information and an uncertain diagnosis of CD, (5) no treatment at PUMCH, (6) no sexual partners or practiced strict contraception, (7) uncertain pathological results staining negative for ACTH and no remission of hypercortisolemia after surgery, or (8) a final diagnosis of ectopic ACTH syndrome.

The diagnosis of CD was based on clinical features, laboratory examinations, sellar magnetic resonance imaging (MRI), BIPSS, and pathological results. The included patients had ACTH-dependent Cushing syndrome, as supported by (A) increased 24-h urinary free cortisol (24-h UFC) or midnight cortisol above 1.8 μg/dL (50 nmol/L); (B) no suppression on low-dose dexamethasone suppression test (LDDST); and (C) morning plasma ACTH within or above the normal range. A reduction rate of >50% in the 24-h UFC level of after high-dose dexamethasone administration (8 mg/day 48-h dexamethasone administration) in the high-dose dexamethasone suppression test (HDDST) was considered suppressed and indicated the possibility of a diagnosis of CD. Sex hormone levels were also measured during hospitalization for surgery.

Dynamic gadolinium–enhanced MRI of the pituitary gland was conducted to identify the source of excessive ACTH production. The presence of pituitary adenoma could be determined by discrete hypointensity on contrast-enhanced imaging or a hypointense region on early-phase dynamic imaging, subsequently becoming less hypointense with the dynamic cycles. A tumor was considered to be a macroadenoma if its maximum dimension was over 10 mm on MRI; otherwise, a microadenoma was predicted. Patients with unsuppressed HDDST, discordant dynamic tests, or equivocal MRI results were recommended to BIPSS. The cutoff value for the inferior petrosal sinus/periphery was 2.0 at baseline and 3.0 after desmopressin stimulation. Some complicated cases were discussed by the pituitary multidisciplinary team (MDT) which consists of experts from departments of endocrinology, neurosurgery, radiology, gynecological endocrinology, ophthalmology, radiotherapy, pathology, and nuclear medicine to determine the subsequent management.

The diagnosis of CD was ultimately confirmed after the pathological immunostaining of resected adenoma was compatible with an ACTH-secreting pituitary adenoma. If pathological results were unclear and revealed negative ACTH staining, the diagnosis of CD was confirmed when the level of morning serum cortisol decreased to <5 μg/dL after surgical treatment.

Overall, CD diagnoses of our patients were based on (1) abnormal indexes in biochemical examinations (A+B+C) and positive BIPSS results or (2) abnormal indexes in biochemical examinations (A+B+C), negative or lack of BIPSS results but positive ACTH immunostaining adenoma, or postoperative morning serum cortisol less than 5 μg/dL. Four patients with negative BIPSS results were recommended for pituitary surgery after discussions of pituitary MDT, and they were confirmed with CD pathologically. Fourteen patients with uncertain pathological results (negative ACTH staining) and no decrease in serum cortisol to less than above criteria were excluded.

### Data Collection

The electronic medical records evaluated at the hospitalization for surgery were reviewed to collect information, including demographics (sex and age), approximate time of and symptoms at time of disease onset, laboratory examination results, imaging and pathology findings, treatment and disease outcomes, number of previous pregnancies, hypertension and diabetes status during pregnancy, and gestational outcomes. The time of disease onset was determined by the occurrence of signs and symptoms of CD (early-onset hypertension, unexpected severe hyperglycemia in gestation, skin thinning, easily bruising, weight gain and proximal weakness, etc.) (13). Symptoms of 19 patients during pregnancy were considered CD-related (weight gain during gestation or postpartum, blood pressure and fasting blood glucose levels, etc.), so these details were accurately recorded in medical charts at surgery and also collected. Since none of patients were screened for CD during pregnancy, there is a lack of biochemical and imaging data on CD diagnosis in this period. Tests for CD in patients with previous pregnancies were applied after their abortions or deliveries. To collect information regarding pregnancy and childbirth, a questionnaire (Supplementary Table 1) was designed and administered online to all female patients through *So jump*, the largest online survey and polling platform in China. The questionnaire included 13 questions about maternal history and neonatal outcomes, such as the types of pregnancy (natural or assisted reproduction), time of previous pregnancies, delivery mode (vaginal or cesarean section), timing of birth (preterm or term) and gender, birth weight (LBW: defined as the weight of newborns <2,500 g at delivery), and complications of the newborns. The time of disease onset, number of previous pregnancies, gestational outcomes, and hypertension and diabetes status during pregnancy were also mentioned in the questionnaire and cross-checked with the medical records.

When details regarding medical history, particularly the timing of disease onset related to pregnancy, could not be confirmed based on medical records or questionnaire feedback, patients were contacted by telephone directly to request additional information and recollections. The reliability and validity of the answer were assessed by an endocrinology expert at PUMCH (LL and HjZ).

### Group Classification

**Figure 1:**
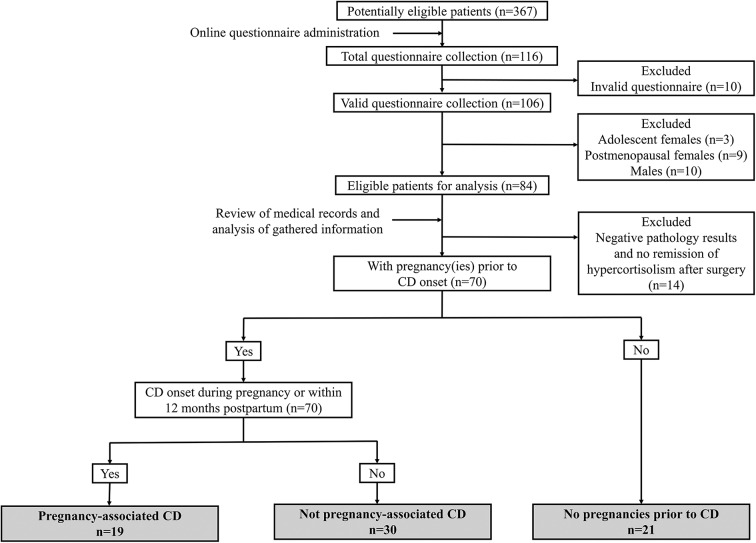
Flow chart of group division and number of patients.

The online questionnaire was directed to potentially eligible participants (*n* = 367) if they (1) received interventions for CD at our hospital during the previous 10 years and (2) keep regular follow-up after being discharged from the hospital and could be contacted for overall review. One hundred and sixteen patients completed questionnaires (31.6%). After the exclusion of invalid questionnaires and those that did not meet the inclusion criteria or met the exclusion criteria, 70 patients were enrolled in our subsequent analysis, and their identities were matched with their identification numbers in their completed questionnaires (Figure 1).

These 70 patients were classified into three subgroups based on questionnaire feedback. (1) The first group comprised women with pregnancy-associated CD (*n* = 19). Pregnancy-associated CD was defined as CD onset potentially related to pregnancy, namely, during gestation or within 12 months after delivery or abortion, and other predisposing factors were excluded according to their own questionnaire feedback (12). The pregnancies of the women in this group all occurred within 12 months prior to disease onset. (2) The second group comprised women with CD that was not associated with pregnancy (*n* = 30). These women had at least one pregnancy before CD onset and an interval between pregnancy and CD onset that was over 12 months. (3) The third group comprised women without pregnancies prior to CD onset (*n* = 21). There were no cases of CD onset followed by pregnancy in our series, probably due to the impairment of fertility and ovulation by the hypercortisolism and concomitant hyperandrogenism of active CD.

### Outcome

Early remission was defined as a serum cortisol concentration ≤5 μg/dL within the first week after surgery. Final remission was defined as remission achieved by multimodal treatment, including repeated surgery, medical treatment, radiation treatment, and/or bilateral adrenalectomy. Recurrence was defined as recurrent hypercortisolism after initial remission. Recurrence time was defined as time to recurrence after initial remission.

### Statistical Analysis

Qualitative data are presented as frequencies and percentages. Quantitative data are presented as means ± standard deviations or medians (ranges). Qualitative data such as treatment, remission, and pregnancy outcomes were analyzed with the χ^2^ test or Fisher's exact test as appropriate. Quantitative data, including age and biochemical data, of the patients were analyzed by the Kruskal–Wallis rank-sum test. Statistical analysis was conducted using SPSS (Version 25.0, IBM SPSS Statistics). A *p* < 0.05 was considered statistically significant.

## Results

### Demographics and Preoperative Clinical Characteristics

Women of reproductive age meeting the inclusion criteria were included, while the remaining individuals (males, adolescent females, menopausal females, and patients with negative pathology results and no remission of hypercortisolism after surgery) were excluded. A total of 70% (*n* = 49) of the included women had at least one pregnancy before CD onset, and of these, 38.8% (*n* = 19) had pregnancy-associated CD (Table 1).

**Table 1:** Demographics and clinical findings of the patients.

	**The relationship with pregnancy**			
	**Pregnancy-associated**	**Not pregnancy associated**	**No pregnancy prior to CD**	***p*-value**
**Demographics**				
Patients (*n*)	19 (27.1%)	30 (42.9%)	21 (30.0%)	
Median of pregnancies prior to CD (*n*)	1 (1–6)	1 (1–3)	**N/A**^**a**^	0.196
Age at last pregnancy prior to disease onset (years)^*^	26.6 ± 3.4	27.0 ± 6.1	**N/A**^**a**^	0.760
Age at initial symptom onset (years)	28.4 ± 4.1	33.3 ± 6.5	24.0 ± 8.1	** <0.001**
Age at diagnosis (years)	31.1 ± 5.6	36.1 ± 6.8	25.4 ± 7.8	** <0.001**
Time to diagnosis (years)	2.7 ± 3.4	2.8 ± 2.7	1.4 ± 1.7	0.142
**Preoperative**				
Morning serum cortisol (μg/dL) (normal range: 4.0–22.3 μg/dL)	26.6 ± 6.5	28.1 ± 13.2	26.1 ± 7.3	0.988
Midnight serum cortisol (μg/dL)	17.8 ± 5.5	18.1 ± 8.3	18.8 ± 7.9	0.852
Morning plasma ACTH (pg/mL) (normal range: <46 pg/mL)	70.1 ± 28.6	65.7 ± 36.0	72.8 ± 46.9	0.869
24 h urine free cortisol (μg) (normal range: <45 μg)	513.1 ± 400.5	445.2 ± 326.5	351.9 ± 228.7	0.272
No suppression on LDDST (*n*)	19 (100.0%)	30 (100.0%)	21 (100.0%)	1
Suppression on HDDST (*n*)	17 (89.5%)	30 (100.0%)	20 (95.2%)	0.108
Testosterone (ng/mL) (normal range: 0–0.75 ng/mL)	0.68 ± 0.38	0.71± 0.30	0.68 ± 0.31	0.881
**Imaging findings and BIPSS**				
**Preoperative**				
Visible tumor (*n*), *N* = 61	16 (84.2%)	24 (80.0%)	21 (100.0%)	0.083
Microadenoma (*n*)	12 (75.0%)	22 (91.7%)	21 (100.0%)	**0.038**
Macroadenoma (*n*)	4 (25.0%)	2 (8.3%)	0 (0.0%)	
BIPSS (*n*), *N* = 38	6 (52.6%)	16 (53.3%)	12 (57.1%)	1
Positive (*n*)	9 (90%)	14 (87.5%)	11 (91.7%)	1
Negative (*n*)	1 (10%)	2 (12.5%)	1 (8.3%)	
**Treatment**				
Number of transsphenoidal surgeries (median)	1 (1–3)	1 (1–3)	1 (1,2)	0.356
Radiation therapy (*n*)	3 (17.6%)	6 (20.0%)	6 (28.6%)	0.703
**Post-operative (in the first week)**				
Morning serum cortisol (μg/dL)	10.5 ± 11.1	8.4 ± 9.9	11.3 ± 15.9	0.796
Ratio of morning cortisol <5 μg/dL	16 (84.2%)	25 (83.3%)	16 (76.2%)	0.793
Median value of morning plasma ACTH (pg/mL)^b^	10.9	11.5	17.4	0.910
Immunostaining for ACTH on pathological examination	17 (89.5%)	29 (96.7%)	21 (100%)	0.348
**Disease outcome**				
Recurrence (*n*)	9 (47.4%)	7 (23.3%)	8 (38.1%)	0.204
Time to recurrence (months)	26.2 ± 18.8	39.7 ± 29.0	22.4 ± 12.6	0.394
Final remission (*n*)	17 (89.5%)	28 (93.3%)	18 (85.7%)	0.669
Follow-up (months)	50.2 ± 27.9	43.4 ± 30.5	48.1 ± 23.2	0.466

a *N/A, data not available*.

b *The post-operative ACTH level was recorded as < 5 pg/mL without a numerical value, thus the plasma ACTH was presented as the median value rather than the mean value*.

* *These data were obtained from questionnaires*.

*Bold value, P < 0.005*.

Regarding the 19 women with pregnancy-associated CD, the mean age at disease onset was 28.4 ± 4.1 years, and the mean age at diagnosis was 31.1 ± 5.6 years, with a mean interval between disease onset and diagnosis of 2.7 ± 3.4 years. There was a significant difference in age at disease onset between the group of women without pregnancy-associated CD and the group of women without pregnancy before CD (*p* < 0.001). The difference in age at disease diagnosis between these two groups was also statistically significant (*p* < 0.001).

When the women with pregnancy-associated CD were compared to those without, the results showed that there were no significant differences among the preoperative biochemical data, including the average levels of morning serum cortisol, midnight serum cortisol, plasma ACTH, 24-h UFC and testosterone, and the results of the LDDST and HDDST. Differences in the postoperative biochemical data including the average levels of morning serum cortisol and morning plasma ACTH were not significant, either. Among the preoperative MRI data, 87.1% (*n* = 61) of the pituitary adenomas were visible, and 90.2% (*n* = 55) of those tumors identified were microadenomas. The rates of microadenomas were 75% in women with pregnancy-associated CD and 100% in women without pregnancy before CD, and the difference was significant (*p* = 0.038). Four patients had negative BIPSS results preoperatively whose response to desmopressin was not verified before they underwent BIPSS with desmopressin stimulation. The ACTH gradient of central/peripheral was 1.09, 1.77, 1.46, and 1.08 at baseline and 2.05, 2.34, 2.87, and 2.26 after DDAVP stimulation for the four patients, respectively. Their diagnoses of CD were confirmed pathologically following surgery.

### Disease Outcome and Remission

A total of 81.4% (*n* = 57) of patients achieved early remission, where a serum cortisol level ≤ 5 μg/dL was detected within 72-h after surgery. Recurrences were documented in 47.4% (*n* = 9), 23.3% (*n* = 7), and 38.1% (*n* = 8) of patients, and the average time to recurrences was 26.2 ± 18.8 months, 39.7 ± 29.0 months, and 22.4 ± 12.6 months for the pregnancy-associated CD group, non-pregnancy-associated CD group, and no pregnancy pre-CD group, respectively. Rates of final remission were 89.5% (*n* = 17), 93.3% (*n* = 28), and 85.7% (*n* = 18) for these three groups, respectively.

### Maternal and Fetal Outcomes

A total of 78 pregnancies among 49 patients were recorded in our analysis. 24% (*n* = 19) of pregnancies, of which 17 (89.5%) occurred spontaneously, were possibly associated with CD onset (Table 2). During the pregnancy period, hypertension and diabetes mellitus in the mothers with pregnancy-associated CD in our setting were detected in 15.7 and 5.2% of patients, respectively. However, hypertension and diabetes mellitus in the mothers with non-pregnancy-associated CD were detected in 13.6 and 3.4% of patients, respectively. There were no significant differences between the two groups.

**Table 2:** Pregnancy-related events in 49 women with and without pregnancy-associated Cushing's disease.

	**Pregnancy-associated CD (*n* = 19)**	**Non-pregnancy-associated CD (*n* = 30)**	***p*-value**
Recorded pregnancies, *N* = 78	*N* = 19	*N* = 59^e^	
Types of pregnancy^*^			
Natural pregnancy	17 (89.5%)	55 (93.2%)	0.630
Assisted reproduction	2 (10.5%)	4 (6.8%)	
**Gestational outcome**			
Induced abortion	0 (0.0%)	5 (8.5%)	0.216
Spontaneous abortion	5 (26.3%)	7 (11.9%)	
Live birth	14 (73.7%)	47 (79.7%)	
Maternal morbidities in pregnancy^**^			
Hypertension	3 (15.7%)	8 (13.6%)	0.826
Diabetes	1 (5.2%)	2 (3.4%)	0.874
Living newborns, *N* = 61	*N* = 14	*N* = 47	
Delivery mode^*^			
Vaginal	11 (78.6%)	26 (55.3%)	0.118
Cesarean section	3 (21.4%)	21 (44.7%)	
Gender of newborn^*^			
Male	6 (42.9%)	27 (57.4%)	0.336
Female	8 (57.1%)	20 (42.6%)	
Timing of birth^*^			
Preterm^a^	4 (28.6%) (30–34 weeks)	4 (8.5%) (32–36 weeks)	0.133
Term	10 (71.4%)	43 (91.5%)	
Fetus complication^*^			
Yes	1 (7.1%)^c^	0 (0.0%)	0.230
No	13 (92.9%)	47 (100.0%)	
Birth weight in all newborns^*^			
Low birth weight^b^	5 (35.7%)	1 (2.1%)	**0.002**
Normal	9 (64.3%)	46 (89.4%)	
Term newborns, *N* = 53^*^	*N* = 10	*N* = 43	
Low birth weight	2 (20.0%)^d^	0 (0.0%)	**0.033**
Others	8 (80.0%)	43 (100%)	

a *Preterm birth was defined as birth of a baby at fewer than 37 weeks of gestational age*.

b *Low birth weight was defined as the weight of newborns <2,500 g regardless of gestational age*.

c *Fetal complication was defined as any abnormalities of newborns reported by mothers, and this child was born with cerebral hypoxia whose mother developed CD after delivery (case 2 in Table 3)*.

d *These two term LBW newborns were found in patients with CD during pregnancy (case 8 and 19 in Table 3)*.

e *These 30 women with a history of pregnancy but without pregnancy-associated CD may be pregnant more than once. Consequently, 59 pregnancies were recorded according to their answering*.

* *These data were obtained from questionnaires*.

** *Data on hypertension and diabetes mellitus in mothers with pregnancy-associated CD were recorded in their hospitalized medical records while those of mothers with non-pregnancy-associated CD were collected from questionnaires*.

*Bold value, P < 0.005*.

The details of the 19 women with pregnancy-associated CD are presented in Table 3. Most of the women were primiparous (*n* = 13). Of the six women with a multiparous obstetric history, one had unexplained recurrent abortions (three spontaneous abortions). Of the 19 pregnancies, five ended with spontaneous abortion, while the other 14 ended with successful live births. All three women who developed symptomatic morbidities during gestation were diagnosed with microadenomas within 12 months postpartum, and of these, two had newly diagnosed hypertension at 16 weeks of gestation and another was diagnosed with severe hypertension (170/140 mmHg) and severe hyperglycemia (fasting blood glucose, FBG: 15 mmol/L) at 29 weeks of gestation. Of the three patients diagnosed with hypertension within 12 months postpartum, two gained more than 30 kg during the gestational period. The other 13 patients presented with less specific signs and symptoms of CD during or immediately after pregnancy, mainly including round face (*n* = 8) and weight gain (*n* = 4). Menstrual irregularity, lactation, acne, and headache were also complaints of these patients. Details of mothers with non-pregnancy-associated CD (*n* = 30) during pregnancy, such as weight gain during gestation or postpartum, blood pressure, and fasting blood glucose levels, were not recorded in medical charts and thereby not collected.

**Table 3:** 19 Patients with pregnancy-associated Cushing's disease.

**No**.	Age at last pregnancy prior to disease onset^*^	**Age at initial symptom onset**	**Predominant signs and symptoms during and after pregnancy**	**Tumor type**	**Tumor size (mm)**	**Recurrent times**	**Disease outcome**	Delivery mode^*^	**Gestational outcome**	Newborn gender^*^	Newborn weight (g)^*^	**Pregnancies prior to CD**	Noteworthy problems during pregnancy^**^
1	22	23	Hypertension (180/110 mmHg)	Macro^a^	11.4	2	Remission	Vaginal	Live birth	F	3,100	6	
			Weight gain^c^ (40 kg in gestation)										
2	28	29	Round face	Micro^b^	6.0	0	Remission	Vaginal	Live birth	F	2,600	2	Fetus: preterm birth (34 weeks), cerebral hypoxia
3	22	22	Weight gain (40 kg during gestation and after delivery)	Macro	16.0	2	Non-remission	Vaginal	Live birth	M	1,600	1	Fetus: preterm birth (31 weeks), low birth weight
4	27	27	Round face	Micro	3.5	1	Remission		Spontaneous abortion	_	_	1	Fetus: spontaneous abortion
5	31	32	Round face	Not seen	_	1	Remission	Vaginal	Live birth	F	1,860	1	Fetus: preterm birth (33 weeks), low birth weight
6	27	28	Headache	Macro	10.2	2	Remission		Spontaneous abortion	_	_	1	Fetus: spontaneous abortion
			Round face										
7	27	28	Round face	Micro	7.3	1	Non-remission	Vaginal	Live birth	M	3,270	1	
8	22	23	Hypertension (130/100 mmHg)	Micro	6.0	0	Remission	C-section	Live birth	F	2,100	1	Mother: hypertension (16 weeks)
													Fetus: low birth weight
9	34	35	Menstrual irregularity	Micro	4.0	0	Remission	Vaginal	Live birth	M	3,500	1	
10	27	27	Acne	Not seen	_	0	Remission		Spontaneous abortion	_	_	3	Fetus: spontaneous abortion
11	27	27	Round face	Micro	3.7	1	Remission	Vaginal	Live birth	F	2,700	1	
12	24	24	Lactation	Micro	3.1	0	Remission	Vaginal	Live birth	F	3,200	1	
13	28	28	Round face	Micro	3.7	3	Remission		Spontaneous abortion	_	_	1	Fetus: spontaneous abortion
			Weight gain (25 kg after abortion)										
14	26	26	Hypertension (170/140 mmHg)	Micro	9.8	1	Remission	C-section	Live birth	F	1,360	1	Mother: hypertension and diabetes mellitus (29 weeks)
			Diabetes mellitus (FBG: 15 mmol/L)										Fetus: preterm birth (30 weeks), low birth weight
15	27	28	Round face	Micro	9.2	0	Remission		Spontaneous abortion	_	_	1	Fetus: spontaneous abortion
			Weight gain (11 kg after abortion)										
16	37	38	Hypertension (160/110 mmHg)	Not seen	_	0	Remission	Vaginal	Live birth	M	3,800	2	
			Weight gain (30 kg in gestation)										
17	33	33	Hypertension (140/100 mmHg)	Micro	8.0	0	Remission	Vaginal	Live birth	F	2,600	2	
18	28	28	Acne	Macro	14.4	0	Remission	Vaginal	Live birth	M	3,250	1	
			Weight gain (20 kg in gestation)										
19	29	29	Hypertension (160/120 mmHg)	Micro	5.1	0	Remission	C-section	Live birth	M	1,600	2	Mother: hypertension (16 weeks)
													Fetus: low birth weight

a *Macro = macroadenoma*.

b *Micro = microadenoma*.

c *Patients complained of sudden or progressive weight gain in the peripartum period despite keeping a healthy lifestyle*.

* *These data were obtained from questionnaires*.

** *The data on maternal complications during pregnancy were recorded in medical records while those on fetal condition were obtained from questionnaires*.

Overall, 61 pregnancies (78.2%) ended with live births. To compare the abnormal pregnancy outcomes among the women with pregnancy-associated CD to those among the women with CD that was unrelated to pregnancy, data regarding live births, spontaneous abortions, maternal and fetal mortality and morbidity, and birth weights of newborns were collected and analyzed. The differences in the rates of spontaneous abortion (26.3 vs. 11.9%) and preterm birth (28.6 vs. 8.5%) between the pregnancy-associated CD group and the non-pregnancy-associated CD group did not reach significance. However, in the pregnancy-associated CD group, the low-birth-weight (LBW) rate was 35.7% (*n* = 5), and in the group of non-pregnancy-associated CD, the LBW rate was 2.1% (*n* = 1); the difference was significant (*p* = 0.002). This significant difference in the rate of LBW infants still remained after four preterm newborns were excluded.

There were four cases of prematurity and five LBW newborns. The weights of newborns ranged between 1,360 and 3,800 g. Three-quarters of the newborns born before 37 weeks of gestation had LBW. The other preterm newborn, weighing 2,600 g, was delivered vaginally and initially maintained in the neonatal intensive care unit due to slight cerebral hypoxia. Her symptoms resolved spontaneously within 72-h after birth. This was the only case of newborn complications reported in our series. There were two term LBW infants in our study. Their mothers developed hypertension during pregnancy and underwent elective cesarean section due to unstable maternal status. The main indication for vaginal delivery was a healthy maternal status and steady labor.

## Discussion

In this study, we investigated the characteristics of pregnancy-associated CD and, for the first time, systematically explored the relationship between pregnancy-associated CD and outcomes of mothers and children. Since infertility was quite common in patients with active CD (4), we could not analyze the maternal and fetal outcomes in patients with preexisting CD before pregnancy. The overall pregnancy outcomes of the group with pregnancy-associated CD were similar to those of the group with normal pregnancy except for a significantly increased risk of LBW. Assessments and close monitoring should be provided to women with suspected symptoms of hypercortisolism such as early-onset hypertension, severe hyperglycemia, a round face, and persistent weight gain, during and after pregnancy.

### Increased Incidence of Pregnancy-Associated CD

A total of 27.1% (*n* = 19) of the women in our study presented with Cushingoid signs during or 12 months postpartum, Based on the National Health Commission of the People's Republic of China (14), the average woman in China had 1.5–1.6 births during our study period. Roughly assuming 92 weeks of gestation (40 weeks) and the following peripartum period (1 year = 52 weeks) per child, the average woman spends 138–147 weeks or 7.0–7.4% of her reproductive years in the gestation and postpartum periods (12). Broder's study on commercially insured patients in the United States from 2009 to 2010 suggested that the highest female CD incidence rates per million person-years were in the 18–24-year-old group, which was around 2.2 times higher than that of females of all ages (15). Thus, it is estimated that the maximum rate of CD in the gestation and postpartum periods is 16.3% (2.2 × 7.4%). However, 27.1% of our patients developed CD during or in a short time after pregnancy, which might possibly indicate the tendency to develop CD and necessarity of tests for CD in the peripartum period.

This phenomenon motivated us to explore the possible reasons for the relatively high rate of pregnancy-associated CD. Pregnancy is characterized by several endocrine biochemical changes, such as an over 100-fold increment of increase in estrogen levels in the blood (16). The effects of estrogen on the human pituitary include stimulation of pituitary angiogenesis, adenohypophyseal hormone secretion, and mitogenesis in specific cell types (17, 18). These proliferative effects involved in pituitary tumor initiation and progression are mediated through nuclear receptors, namely, ERα and ERβ (19). A positive expression of ERα and ERβ has been shown in multiple subtypes of pituitary adenoma, including growth hormone, prolactin, follicle-stimulating hormone/luteinizing hormone, and ACTH-secreting adenomas and null cell adenomas (1, 18). This expression pattern of ER may be related to a higher proportion of women with peripartum CD onset. Further bench and beside investigations are needed to delineate the precise mechanisms of actions of estrogen on pituitary ACTH-secreting adenomas.

Alternatively, the observed predominance of CD among postpartum women may be attributed to the activation of the HPA axis during pregnancy and the immediate postpartum period (12). Placental production of corticotropin-releasing hormone (CRH), hypersensitization of corticotrophs to CRH, and desensitization to negative feedback by cortisol contribute to the surge of maternal plasma ACTH and cortisol levels. This increased stimulation of pituitary corticotrophs may also stimulate pituitary tumorigenesis, accounting for the occurrence of peripartum CD (9).

It should be noted that 30% of women in our cohort had no pregnancies prior to CD onset. This fact indicated that physiological changes related to pregnancy may just be one of the potential factors among many factors that ultimately contribute to the development of a pituitary corticotroph adenoma. Also, given the limited sample size, the proportion of pregnancy-associated CD might be a result of chance and related to the natural course of disease, irrespective of pregnancy.

### Maternal and Fetal Outcomes in the Pregnancy and Non-pregnancy-Associated CD

The rates of hypertension, diabetes, spontaneous abortion, and preterm birth trended higher in the pregnancy-associated CD group in this study, although the differences among groups were not significant. Compared with those of 1,102 healthy pregnant women in Beijing, most of the rates of maternal morbidities and adverse fetal outcomes except for the rate of diabetes in the pregnancy-associated CD group are also relatively increased (hypertension: 15.7 vs. 5.0%; diabetes: 5.2 vs. 13.1%; spontaneous abortion: 26.3 vs. 19.4%; preterm birth: 21.1 vs. 19.9%) (20). In previous studies involving pregnant women with active Cushing's syndrome, the rates of hypertension, diabetes mellitus, spontaneous abortion, and preterm birth were 40.5, 36.9, 23.6, and 66%, respectively, which were all much higher than those in our study (5).

This discrepancy regarding maternal and fetal complications may have a multifactorial explanation. Firstly, this study focuses on patients gradually developing CD in the peripartum period while most previous cases were patients with preexisting tumors that had already progressed to overt CD during pregnancy. Moreover, the etiology primarily accounting for the presence of Cushingoid features varies markedly across studies. Specifically, nearly half of the previously identified cases of hypercortisolism in pregnancy had adrenal-dependent disease (16), whereas all the cases in our paper were pituitary in origin. Given that disease originating in the pituitary is less aggressive, it is predictable that our patients would have better outcomes (5, 21, 22). In addition, previous studies were systematic reviews in which data were collected from sporadic cases ranging widely in time period and region. Different regional centers defined pregnancy complications per their own unique criteria, which may have introduced statistical bias and lessened the power of the data. Thus, typical symptoms or complications of CD maybe rarely occurred in our patients or their children.

In our study, there were no significant differences in pregnancy outcomes, except the LBW newborn rate, between the group with pregnancy-associated CD and the group without pregnancy-associated CD. LBW newborns, secondary to intrauterine growth restriction or premature birth, accounted for 35.7% (*n* = 5) of infants born to mothers with pregnancy-associated CD, among whom the proportion of LBW was significantly increased compared to that among the control subjects. This proportion is also higher than the reported incidence of LBW (8.1%) in mainland China over the past 10 years (23). There were two term LBW infants in the group with pregnancy-associated CD, and both of their mothers had newly diagnosed hypertension at 16 weeks of gestation. Another mother complicated by hypertension and severe diabetes (FBG: 15 mmol/L) at 29 weeks of gestation, delivered a LBW newborn at 30 weeks. Although the rate of hypertension was not significantly different among groups, it trended higher in the pregnancy-associated CD group and the possibility that hypertension increased the risk of LBW could not be ruled out. Thus, appropriate biochemical tests to rule out a secondary etiology including hypercortisolism might be indicated in women who have early-onset hypertension (before 20 weeks of gestation) and (or) severe hyperglycemia not compatible with gestational diabetes to decrease the risk of adverse pregnancy outcomes.

Few previous reports have documented the profiles of pregnancy-associated CD and fetal birth weight. In a case in which the patient's CD symptoms, including hyperpigmentation, rapid weight gain, fatigue, and weakness, began in the third trimester and whose symptoms met our proposed criteria for pregnancy-associated CD, a healthy full-term infant with a marginally low birth weight of 2,500 g was reportedly delivered (24). The notion of an increased LBW risk with pregnancy-associated CD is supported by another uncomplicated case of a healthy male infant weighing 2,100 g upon delivery at 34 weeks whose mother progressively developed Cushingoid symptoms during pregnancy (25). The significantly increased risk of LBW in mothers with pregnancy-associated CD in our setting may be due to the impact of underlying occult hypercortisolism during the pregnancy. However, this difference could be detected only by chance and has to be verified in large-scale studies. Underlying factors that govern the potential association between pregnancy-associated CD and LBW require further investigation.

### Difficulties in Management of Pregnancy-Associated CD

CD is known to be a slow-progressing disease that is often clinically atypical in the early stage. For patients with overt CD onset during pregnancy and postpartum, there are two possibilities for the development of CD: First, event dormant or small slow-growing pituitary corticotroph adenomas have been present before which might be aggravated or accelerated by pregnancy; second, the pregnancy might somehow directly promote the occurrence of ACTH-secreting pituitary adenoma in some patients leading to a high risk of CD following pregnancy. The earlier the symptoms of CD are noticed during clinical therapeutics, the better the prognosis for both mother and fetus. Considering the relatively high incidence of pregnancy-associated CD, clinicians should pay more attention to this kind of disease during the peri-partum period.

The clinical signs of CD are not sufficiently specific during pregnancy to allow for a certain and early diagnosis, and the active disease state of CD during pregnancy may be easily ignored due to its rarity (26, 27). A round face and weight gain were frequent presentations in the pregnancy-associated CD group, which were not specific enough during pregnancy to lead to a diagnosis of CD. However, because these symptoms and signs did not resolve or gradually became apparent during the postpartum period, CD could eventually be diagnosed. As such, assessments and close monitoring should also be provided to women who present with persistent suspicious CD symptoms (hypertension, diabetes, round face, weight gain, etc.) during and following pregnancy.

For the management of pregnancy-associated CD, those diagnosed during pregnancy are more difficult to treat since there were no guidelines available so far. Since CD manifests with variable severity, treatment should be provided appropriately varying from conservative approaches in mild hypercortisolemia to medical treatment or surgical intervention in severe cases to lower the risk of maternal and fetal morbidity (28). If the diagnosis of CD is confirmed during the gestational period and the decision to perform an intervention is reached, TSS could be the treatment of choice in the second trimester (29). For those who are not eligible for surgery or has failure of surgery, medical treatment should be cautiously used to achieve better outcomes of CD and pregnancy (6, 30). Cabergoline seems to confer good control of hypercortisolism during pregnancy (7, 8). Metyrapone may be indicated late in the third trimester (29). Ketoconazole is considered an emergency pharmacological therapeutic option because in rats teratogenicity was demonstrated (8). Other medical treatments including mifepristone and mitotane are not recommended due to potential teratogenicity and embryotoxicity to the fetus (7, 8).

### Limitations

This study has several limitations. First, it did not help to clarify the underlying mechanism involved the potential association between CD and pregnancy due to its retrospective and descriptive nature. Second, the pregnancy information was collected from online questionnaires. Although some key information including the timing and symptoms of disease onset, number of previous pregnancies, and maternal complications obtained from the questionnaires was cross-checked with data from medical charts, details regarding pregnancy that relied on patients' recollection may have caused inevitable bias. Third, there may be a risk of overlap between patients with pregnancy-associated CD and those with non-pregnancy-associated CD. These patients were not tested for CD (ACTH or serum cortisol level, etc.) during pregnancy and their diagnoses of CD were postponed postpartum, so the accurate time of hypercortisolism occurrence cannot be determined (31). These women with non-pregnancy-associated CD probably underwent a long period of subclinical hypercortisolism as pregnancy advances and appeared apparent Cushingoid couple of years following deliveries. In an ideally designed study, medical records that include biochemical and imaging data during pregnancy, complete data on all maternal morbidities, and birth records should be collected and analyzed. Another limitation is the relatively small sample size because CD is a rare disease. The results of this study need to be further validated with basic experiments and wide-scale studies of women with CD to improve the generalizability of our findings.

## Conclusion

In this retrospective study, 27.1% of women at reproductive age with CD developed CD during or soon after pregnancy, which might be induced by the hormonal milieu of pregnancy. Pregnancy-associated CD was related to an increased risk of LBW newborns; however, large-scale studies are needed to determine the relationship between this disease and other abnormal pregnancy outcomes. High clinical suspicion and close monitoring for CD should be provided to women in the peripartum period, especially those presenting with suspected symptoms of CD such as early-onset hypertension, severe hyperglycemia, and persistent weight gain, during and following pregnancy. Postpartum follow-up and examinations may be applied in those with diseases diagnosed as being related to gestation that remain active during the postpartum period.

## Data Availability Statement

The datasets generated for this study are available on request to the corresponding author.

## Ethics Statement

The studies involving human participants were reviewed and approved by institutional review board of Peking Union Medical College Hospital, Chinese Academy of Medical Sciences. Written informed consent to participate in this study was provided by the participants' legal guardian/next of kin. Written informed consent was obtained from the individual(s) for the publication of any potentially identifiable images or data included in this article.

## Author Contributions

KT wrote the main manuscript text and prepared figures and tables. LL, MF, HlZ, KC, RW, XS, HjZ, and ZL collected and analyzed the data. LL and MF designed the work and critically revised it for important intellectual content. All authors reviewed the manuscript and approved it for publication.

## Conflict of Interest

The authors declare that the research was conducted in the absence of any commercial or financial relationships that could be construed as a potential conflict of interest.
