# Supplementary material for: The Incidence of Pregnancy-Associated Cushing's Disease and Its Relation to Pregnancy: A Retrospective Study
Source: Front Endocrinol (Lausanne). 2020 May 29;11:305. doi: 10.3389/fendo.2020.00305 (PMC7272570; doi:10.3389/fendo.2020.00305)
Supplement: Supplementary file 1 [file Table_1.DOCX]

Supplementary Material

**Supplementary Table 1. Questionnaire to investigate the association between Cushing’s disease and pregnancy**

| The purpose of this questionnaire is to provide information about association between pregnancy and Cushing’s disease. Your answer is of great value to our research. It is expected to spend you 15 minutes completing the questionnaire. Please provide accurate information and answer the questions to the best of your ability unless you are requested to skip over a question. | |
| --- | --- |
| 1. Your medical number of Peking Union Medical College Hospital (PUMCH) |  |
| 1. Gender | ○Male ○Female |
| 1. Living place |  |
| 1. Home place |  |
| 1. Date of birth |  |
| 1. Time of onset of Cushingoid features, including hypertension, facial rounding, dorsocervical fat, unexplained weight gain, edema, etc |  |
| 1. Time of diagnosis of Cushing’s disease |  |
| 1. Time of transphenoidal surgical treatment at PUMCH |  |
| 1. Have you received radiation therapy at PUMCH? If yes, please fill the accurate time. |  |
| 1. Have you ever experienced disease recurrence? If yes, please fill the accurate time. |  |
| 1. Disease outcome | ○Active disease ○Remission |
| Following questions for female patients | |
| 1. Have you ever been pregnant? | ○Yes ○No |
| 1. Times of previous pregnancy(s) |  |
| 1. Accurate time of each pregnancy (beginning-ending) |  |
| 1. Conception method of each pregnancy |  |
| 1. Gestational outcome of each pregnancy |  |
| 1. Delivery mode of each pregnancy |  |
| 1. Gender of newborn (s) |  |
| 1. Birth weight of newborn (s) |  |
| 1. Complication of newborn (s) |  |
| 1. Is your symptom onset of Cushing’s disease before, during or after delivery? | ○Before ○During ○After |
| 1. Is your symptom onset of Cushing’s disease during pregnancy or within 1 year postpartum? | ○Yes ○No |
| 1. Do you develop hypertension during pregnancy? If so, did your symptom resolve spontaneously after delivery? |  |
| 1. Do you develop diabetes mellitus during pregnancy? If so, did your symptom resolve spontaneously after delivery? |  |
